# Longitudinal Analyses of the Reciprocity of Depression and Anxiety after Traumatic Brain Injury and Its Clinical Implications

**DOI:** 10.3390/jcm10235597

**Published:** 2021-11-28

**Authors:** Biyao Wang, Marina Zeldovich, Katrin Rauen, Yi-Jhen Wu, Amra Covic, Isabelle Muller, Juanita A. Haagsma, Suzanne Polinder, David Menon, Thomas Asendorf, Nada Andelic, Nicole von Steinbuechel, CENTER-TBI Participants and Investigators

**Affiliations:** 1 Institute of Medical Psychology and Medical Sociology, University Medical Center Goettingen, Waldweg 37A, 37073 Goettingen, Germany; marina.zeldovich@med.uni-goettingen.de (M.Z.); yi-jhen.wu@med.uni-goettingen.de (Y.-J.W.); amra.covic@med.uni-goettingen.de (A.C.); isabelle.mueller@med.uni-goettingen.de (I.M.); nvsteinbuechel@med.uni-goettingen.de (N.v.S.); 2 Division of Psychology and Language Sciences, University College London, London WC1H 0AP, UK; 3 Department of Geriatric Psychiatry, Psychiatric Hospital Zurich, University of Zurich, Minervastrasse 145, 8032 Zurich, Switzerland; katrin.rauen@uzh.ch; 4 Institute for Stroke and Dementia Research (ISD), University Hospital, LMU Munich, Feodor-Lynen-Straße 17, 81377 Munich, Germany; 5 Department of Public Health, Erasmus MC, University Medical Center Rotterdam, 3000 CA Rotterdam, The Netherlands; j.haagsma@erasmusmc.nl (J.A.H.); s.polinder@erasmusmc.nl (S.P.); 6 Department of Emergency Medicine, Erasmus MC, University Medical Center Rotterdam, 3000 CA Rotterdam, The Netherlands; 7 Division of Anaesthesia, University of Cambridge/Addenbrooke’s Hospital, Box 157, Cambridge CB2 0QQ, UK; dkm13@cam.ac.uk; 8 Department of Medical Statistics, University Medical Center Goettingen, 37073 Goettingen, Germany; thomas.asendorf@med.uni-goettingen.de; 9 Department of Physical Medicine and Rehabilitation, Oslo University Hospital, 0424 Oslo, Norway; NADAND@ous-hf.no; 10 Research Centre for Habilitation and Rehabilitation Models and Services (CHARM), Faculty of Medicine, Institute of Health and Society, University of Oslo, 0373 Oslo, Norway

**Keywords:** major depression, generalized anxiety disorder, traumatic brain injury, longitudinal, reciprocal relationship

## Abstract

Depression and anxiety are common following traumatic brain injury (TBI). Understanding their prevalence and interplay within the first year after TBI with differing severities may improve patients’ outcomes after TBI. Individuals with a clinical diagnosis of TBI recruited for the large European collaborative longitudinal study CENTER-TBI were screened for patient-reported major depression (MD) and generalized anxiety disorder (GAD) at three, six, and twelve months post-injury (N = 1683). Data were analyzed using autoregressive cross-lagged models. Sociodemographic, premorbid and injury-related factors were examined as risk factors. 14.1–15.5% of TBI patients reported moderate to severe MD at three to twelve months after TBI, 7.9–9.5% reported GAD. Depression and anxiety after TBI presented high within-domain persistency and cross-domain concurrent associations. MD at three months post-TBI had a significant impact on GAD at six months post-TBI, while both acted bidirectionally at six to twelve months post-TBI. Being more severely disabled, having experienced major extracranial injuries, an intensive care unit stay, and being female were risk factors for more severe MD and GAD. Major trauma and the level of consciousness after TBI were additionally associated with more severe MD, whereas being younger was related to more severe GAD. Individuals after TBI should be screened and treated for MD and GAD early on, as both psychiatric disturbances are highly persistent and bi-directional in their impact. More severely disabled patients are particularly vulnerable, and thus warrant timely screening and intensive follow-up treatment.

## 1. Introduction

Worldwide, more than 50 million people experience a traumatic brain injury (TBI) every year, and it is estimated that about half the world’s population will suffer at least one TBI over their lifetime [1]. Depression and anxiety are the most frequently observed mental health disorders after TBI [2,3,4]. Some plausible explanations of the emergence of post-injury depression and anxiety include the fear of higher mortality rate [5], heightened stress reactivity and enhanced fear sensitivity [6], disruption of neural circuits [7], and post-traumatic changes of neurotransmitters in the brain [8]. A large meta-analysis reported that 27% of patients were clinically diagnosed with depression following TBI and 38% of clinically significant levels of depression assessed with patient-reported outcome measures (PROMs) [9]. A further meta-analysis on anxiety reported that around 11% of patients after TBI were diagnosed with a generalized anxiety disorder (GAD) and 37% reported clinically significant levels of anxiety according to PROMs [10]. The neglect and undertreatment of such psychiatric problems is reported to hamper patients’ recovery, interfere with their mental health [11], and elevate the risk of a long-term chronic health condition [12], poorer social functioning [13], lower quality of life [14,15], and increased health-care costs [16].

In general, post-traumatic depression and anxiety have been reported in a large body of literature [2,17,18,19,20,21,22]. They are either treated as two subfactors of a general underlying distress factor [23] assessed using composite scales [24,25], or as two distinct diseases diagnosed by means of the International Classification of Diseases (ICD-10) [26] or Diagnostic and Statistical Manual of Mental Disorders (DSM-5) [27], or separate scales, requiring differential diagnosis and treatment. Longitudinal investigations of coexisting depression and anxiety after TBI are scarce and dominated by measuring their prevalence. A limited number of studies have attempted to address the joint developmental patterns of depression and anxiety post TBI: Barker-Collo et al. [28] have reported multiple trajectories for patient-reported anxiety and depression across a four-year time span in individuals after mild TBI, whereby the largest proportion of respondents experienced anxiety and depression within the first year post-injury. Using a group-based trajectory model, Ren et al. [29] have demonstrated strong associations between continuously high symptoms of patient-reported depression and anxiety in individuals after severe TBI within two years post-injury. A few studies used the cross-lagged panel design to investigate the reciprocity of the two conditions, focusing on the temporal relationship between functional disability and mental health while assessing patient-reported depression and anxiety separately [30] or jointly [31]. However, the longitudinal differential interplay between depression and anxiety post-TBI, once their persistence and coexistence have been accounted for, remains unclear. The high rates and the risk of psychiatric disturbance occurring within the first year after TBI demonstrated by previous studies [2,32] suggest that a better understanding of their reciprocity in this period could shed light on the causal relationship between these two domains. Such knowledge may inform patients, caregivers, and professionals on targeted interventions, health management, and prevention strategies.

In addition, the evidence as to which factors are associated with higher levels of depression and anxiety following TBI is inconsistent [33] and limited by small sample sizes [34,35,36,37]. Investigations into the impact of extracranial injuries on depression and anxiety in individuals after TBI are scarce, for example. To our knowledge, only one study has investigated this topic, concentrating on patient-reported depression at six months post-injury [38]. The comprehensive exploration of potential risk factors will further optimize early screening, support vulnerable patients, and allow suitable participants to be recruited for interventional/clinical trials.

The current study therefore aims (1) to explore the reciprocal relationship between patient-reported major depression (MD) and GAD over time and (2) to examine risk factors for more severe perceived depression and anxiety after TBI.

## 2. Methods

### 2.1. Participants

The participants included in these analyses were part of the prospective longitudinal observational study of Collaborative European NeuroTrauma Effectiveness Research in Traumatic Brain Injury (CENTER-TBI) [39,40]. Data were extracted from the CENTER Core 2.0 dataset. The core study comprised 4509 patients with a clinical diagnosis of TBI, who were enrolled at 63 different medical centers in 17 European countries and Israel. Participants had a clinical diagnosis of TBI and a clinical indication of a cerebral computed tomography (CCT), presented within 24 h of the injury and provided their informed consent, obtained in line with local and national requirements, and displayed no severe pre-existing neurological disorders [39]. The participants included were assigned to one of three clinical care pathways: admitted to the Emergency Room (ER), to a hospital ward (ADM), or to the intensive care unit (ICU). Data collection was carried out either at the hospital, through face-to-face or telephone interviews, or via postal mail. Further details have been published elsewhere [40]. The current study included participants who had filled in PROMs concerning depression and anxiety screening for at least two time points of the three, six and twelve months assessments after TBI. Individuals with premorbid psychiatric problems were excluded from the analyses to avoid a confounding bias.

### 2.2. Ethical Approval

The CENTER-TBI study was conducted in conformance with all relevant local national ethical guidelines and regulatory requirements for recruiting human subjects, as well as with relevant data protection and privacy regulations, and patients gave their informed consent. The study obtained ethical clearance from the relevant institutions both in the EU and in all the countries involved in the project (for a list of sites, ethical committees, and ethical approval details, see https://www.center-tbi.eu/project/ethical-approval accessed on 4 November 2021).

### 2.3. Measures

Patients’ sociodemographic information, including their age, sex, employment status, relationship status, education level, as well as details of their clinical pathway and pre-injury psychiatric problems, was collected at baseline. The Glasgow Coma Scale (GCS) was used to characterize the level of consciousness and severity of TBI [41]. Major trauma was measured using the Abbreviated Injury Scale (AIS) [42] and defined as positive for Injury Severity Scores (ISS) ≥16 [43]. Major extracranial injury (MEI) was used to categorize major injury in the non-head and neck regions [44] and defined as positive when AIS items in those regions displayed scores ≥3. Functional disability and recovery was rated using the Glasgow Outcome Scale—Extended (GOSE) [45] after TBI. For further details, see Online Supplemental Notes 1.1–1.2.

The Patient Health Questionnaire-9 (PHQ-9) is a patient-reported MD screener with nine items based on the DSM-IV criteria [46] using a four-point Likert scale indicating no symptoms (0 = not at all) to MD symptoms nearly every day (3 = nearly every day) and a two-week frame of reference. The sum score (0 to 27) comprises all items. PHQ-9 scores of 5, 10, 15, and 20 indicated mild, moderate, moderately severe, and severe depression [46].

The General Anxiety Disorder-7 (GAD-7) [47] measures patient-reported GAD based on DSM-IV criteria with seven items on a four-point Likert scale (0 = not at all to 3 = nearly every day) and a two-week frame of reference. The sum score comprises all items. GAD-7 scores of 5, 10, and 15 indicated mild, moderate, and severe levels of anxiety [47].

### 2.4. Statistical Analysis

Patient-reported MD and GAD at three, six, and twelve months after TBI, as well as potential risk factors, were examined descriptively (see Online Supplemental Notes 2.1) and then as unobserved latent variables in a structural equation modelling (SEM) framework [48]. Their longitudinal measurement invariance (MI) was tested as a prerequisite for the following analyses (see Online Supplemental Notes 2.2) and their longitudinal reciprocity was examined using autoregressive cross-lagged (ARCL) models [49,50,51]. Here, autoregressive (AR) effects describe the impact of a construct on itself measured at a later time point, whereby larger AR coefficients indicate little inter-individual variance from the previous time point, i.e., greater stability [52]. Cross-lagged (CL) effects also describe the influence of one construct on another measured at later time points, after controlling for their concurrent and AR effects. A series of nested ARCL models were compared with multiple model fit criteria to identify the best fit with the data (see Online Supplemental Notes 2.3).

Potential risk factors were separately added to the optimal ARCL model as covariates, to investigate how they were associated with the level of MD and GAD. Two types of factors were examined: sociodemographic (i.e., age, sex, employment status, relationship status, and education level) and injury-related (i.e., clinical pathway, GCS, MEI, major trauma, and GOSE) (see Online Supplement 2.4).

Longitudinal tests of MI and ARCL models were conducted using Mplus, version 7.3 [53]. The weighted least square mean and variance adjusted (WLSMV) estimation dealt with categorical data [54] and full information maximum likelihood estimation with missing data. Descriptive analyses were carried out using R version 3.6.1 [55]. A two-tailed *p* < 0.050 was considered statistically significant. Multiple testing was adjusted using a Bonferroni correction, in which the significance threshold (0.050) is divided by the number of tests performed.

### 2.5. Role of the Funding Source

The funder of the study had no role in study design, data collection, data analysis, data interpretation, or the writing of the report. The corresponding author had full access to all the data in the study and had final responsibility for the decision to submit for publication.

## 3. Results

### 3.1. Descriptive Statistics

**Figure 1:**
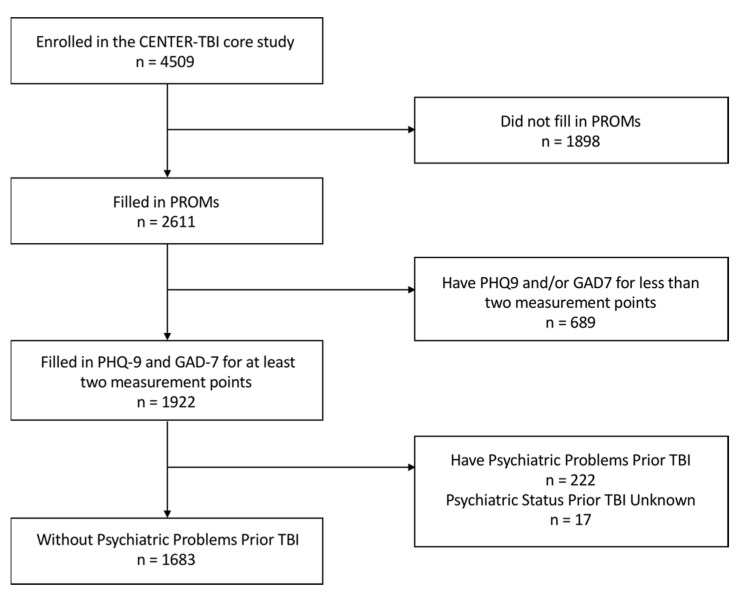
Attrition flowchart. PROM = patient-reported outcome measure; PHQ-9 = Patient Health Questionnaire-9; and GAD-7 = General Anxiety Disorder-7.

The effective sample consisted of 1683 participants (see Figure 1 for a flowchart). Compared to this sample, those excluded from the study were more frequently unemployed, not in a stable relationship, less educated, recruited in the ER, more severely disabled, and reported higher levels of depression and anxiety after TBI (Online Supplemental Tables S1 and S2). If the 928 participants excluded, 34.3% had psychiatric problems prior to the TBI (Online Supplemental Table S3).

The sociodemographic characteristics are presented in Table 1. More individuals in the sample were male (66.5%), middle-aged or older (age M = 49.25, SD = 19.55), educated (years of education M = 13.89, SD = 4.13), employed (58.0%), and in a stable relationship (56.5%). Proportionally fewer individuals (19.0%) were from the ER, 40.3% from the ADM, and 40.7% from the ICU sample. The unbalanced distribution across clinical pathways was to some extent design-related, since the study design required patients admitted to the ER to be assessed at three and six months, but not at twelve months after TBI (see Table S4 for detailed response rates). About 11.7% had experienced psychiatric problems before the TBI (Online Supplemental Table S4). Half of the sample had a major trauma (50.1%), the majority of the participants sustained a mild TBI (76.6%), had no MEI (69.7%), and displayed good recovery six months after TBI (66.2%). 

Patient-reported symptoms of MD and GAD are shown on the scale level in Table 2 and on the item level in Online Supplemental Table S5. Among all participants, mean scores of MD decreased significantly from three to six months after TBI (*t_3m–6m_*(1435) = 3.98, *p* < 0.0001) and remained stable after that (*t_6m–12m_*(1072) = −0.22, *p* = 0.83). Similar patterns were found for GAD over time (*t_3m–6m_*(1431) = 2.09, *p* = 0.036; *t_6m–12m_*(1077) = 0.24, *p* = 0.81). Overall, 23.3% and 14.1% of the participants reported moderate to severe MD and GAD at least at one time point within twelve months after TBI. The prevalence of having moderate to severe levels of psychiatric problems at three, six, and twelve months was 15.5%, 14.1%, and 15.5% for MD (i.e., PHQ-9 score ≥ 10) and 9.5%, 7.9%, and 8.0% for GAD (i.e., GAD-7 score ≥ 10), respectively. The prevalence of different severity levels of MD and GAD (i.e., mild, moderate, moderately severe, and severe) for to different severity/cut-offs is shown in Table 2. Patients with and without concomitant MEI showed no significant difference in the rate of moderate to severe MD or GAD (Online Supplemental Table S6). Limited information was available concerning corresponding treatment during rehabilitation. Data concerning the use of psychological services were collected for only 22.9% of the participants (Online Supplemental Table S7). Among patients with moderate to severe MD or GAD at any time point after TBI, less than 40% received any psychological services during rehabilitation.

### 3.2. Longitudinal Cross-Lagged Model

**Figure 2:**
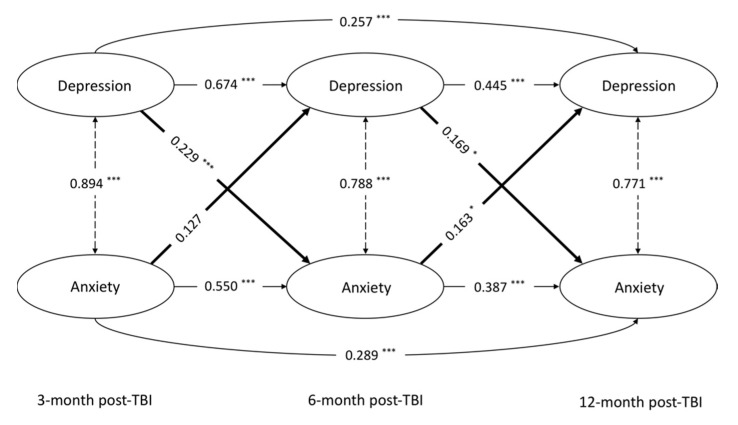
Reciprocal relations between depression and anxiety three to twelve months after TBI stated by model 3 (N = 1683). Cross-sectional correlations between depression and anxiety are shown in dotted lines. Autoregressive and cross-lagged effects are shown in solid and bold lines, respectively. All estimates are standardized. This model accounted for 62.4% and 66.0 of total variance in depression at six and twelve months after TBI, and 58.0% and 62.0% of total variance in anxiety at six and twelve months after TBI. *** *p* < 0.001, and * *p*< 0.05.

As shown in Table 3, the longitudinal MI of the latent constructs of MD and GAD was supported by formal testing (Online Supplemental Notes 3.1). The ARCL model with the best fit is presented in Figure 2 (see Table 4 and Online Supplemental Notes 3.2 concerning model selection; see Online Supplemental Table S8 concerning latent structure). Cross-sectional correlations between MD and GAD at all time points were high despite a significant decrease over time (χ^2^(1) = 343.867, *p* < 0.001). High persistency (AR pathways) was found in both disorders after TBI. The impact remained at high levels between adjacent time points (three to six months vs. six to twelve months) and was statistically stable over time (χ^2^ (1) = 3.344, *p* = 0.068 for MD; χ^2^ (1) = 2.405, *p* = 0.12 for GAD), while those between distant time points were lower. Cross-lagged pathways from MD to GAD were both significant from three to six months and from six to twelve months, indicating that MD at an earlier time point was associated with GAD at a later time point. The extent of such impacts remained at a similar level over time (χ^2^ (1) = 0.290, *p* = 0.59 for MD). In contrast, CL pathway from GAD to MD were only significant from 6 to 12 months after TBI.

### 3.3. Factors Associated with Higher Levels of MD and GAD after TBI

Potential risk factors for MD and GAD levels after TBI were added into the ARCL model separately as baseline covariates (Table 5). Including covariates did not affect the model fit or the existing pathways of the final model. Being female, having stayed at an ICU, having MEI or poorer functional recovery at six months after TBI were strongly associated with more severe MD and GAD after TBI even after Bonferroni corrections. Being without a stable relationship, having more severe injury and major trauma were associated with increased levels of MD only. In contrast, being younger was associated with more GAD. Employment status and years of education showed no predictive value for the level of MD and GAD after TBI.

## 4. Discussion

To our knowledge, this study presents the first attempt to explore the interplay between MD and GAD within twelve months across all TBI severity groups. Our findings demonstrate a bi-directional association between these two psychiatric disorders throughout this time period.

The levels of both MD and GAD decreased among individuals after TBI during the first year post-injury, which is in line with previous studies [28,29]. However, when the prevalence of moderate to severe MD was inspected, an increase was seen from six to twelve months after TBI, which may reflect a later onset of more chronic MD symptoms in the developmental course of MD during the first year after TBI [56]. The rates of moderate to severe MD and GAD were lower than those reported in recent meta-analyses [9,10], which may be due to the assessment tools and the application of stricter cut-offs. Nonetheless, our considerable moderate to severe rates of MD and GAD warrant paying closer attention to these problems. Whether one views them as outcomes after experiencing TBI or as determinants of the outcome, they may hamper the recovery process. Interventions tackling MD and GAD may mitigate adverse effects on functional recovery as well as the psychological health of those affected.

Despite the substantial proportion of patients suffering from MD and/or GAD symptoms after TBI, only very few of them received psychological support or therapy during rehabilitation, with data on interventions unavailable in over three quarters of the patients. This gap between needs and supply in terms of assessing patients, diagnosis of anxiety and depression and providing therapy, calls for a better monitoring of the psychiatric status and psychological functioning of individuals after TBI.

MD and GAD after TBI had moderate stability and strong concurrent associations, which is in line with previous research on the persistence of psychiatric problems [29,57] and the frequently reported coexistence of depression and anxiety following TBI [2,17,18,19,22].

Together, these findings emphasize the importance of early diagnosis and interventions. MD and GAD should be assessed at the earliest convenience after TBI and they should always be screened together. In the event of one such problem already being confirmed, patients should be screened for the other problem without undue delay. Patients found to be positive should be further assessed by clinicians using standardized interviews to obtain a differentiated robust diagnosis. Medical treatment of MD and GAD should follow as soon as possible after the diagnosis.

MD and GAD should also be assessed longitudinally. MD at three and six months after TBI significantly impacted patient-reported GAD at a later adjacent time point, i.e., at six and twelve months after TBI. In contrast, GAD-driven impacts were only significant for GAD at six months after TBI influencing MD at twelve months after TBI. These cross-lagged effects are over and above the within-domain persistence of MD and GAD and concurrent associations between MD and GAD. Therefore, these findings may help to determine the target of potential interventions at different stages after TBI. Considering depression at three months after TBI which leads to both, later depression and anxiety, we suggest that treatment after TBI may firstly focus on depression. In contrast, from six months after TBI onwards, equal attention should be paid to depression and anxiety as then they begin to have bidirectional impacts on each other.

Taken together, MD and GAD after TBI display an interplay of comorbid psychiatric and psychological problems. The decreased stability of MD and GAD from six to twelve months post-TBI (relative to three to six) sheds light on the potential of individual resilience, highlighting the importance of targeting this to promote psychological as well as functional recovery during and after rehabilitation [58,59,60]. Treatment programs, such as Cognitive Behavioral Therapy (CBT), mindfulness-based interventions, or Acceptance and Commitment Therapy (ACT), may be helpful in coping with MD and GAD and prevent persisting affective disorders.

Our results are consistent with the acknowledged vulnerability of females for anxiety and depression [61,62]. We were able to identify the protective role of being in a stable relationship for decreasing the risk of developing MD after TBI, which had not been revealed by previous research [34,63]. Poorer functional recovery was associated with more severe MD and GAD after TBI, which is consistent with previous findings [37,64]. However, previous evidence from cross-lagged models suggests that functional disabilities have limited temporal influence on mental health problems beyond cross-sectional associations [30,31].

Individuals with concomitant MEI displayed increased levels of MD and GAD after TBI. Our findings add to the scarce knowledge about the impact of mental health problems after TBI in individuals with MEI, who are very often excluded from studies on outcomes after TBI to avoid bias [65]. Until recently, only Carroll et al. [38] had rigorously investigated the association between depression and MEI after TBI and found that in patients with mild TBI, those with additional MEI were more likely to suffer from moderate to severe depression compared with those with isolated TBI. The association between MEI and elevated levels of GAD in our study adds to this knowledge and echoes mouse model findings reporting that anxiety-related behavior appears in mice after TBI with MEI but not in mice after isolated TBI [66]. Taken together, our findings suggest that anxiety after TBI may be caused by concomitant injury in non-head and neck regions rather than TBI itself. This hypothesis may explain our finding on the level of consciousness and major trauma after TBI, that individuals with more severe TBI and major trauma suffered from increased depression but not anxiety. Another finding supporting this is that patients from ICUs, a clinical pathway for which we assume that patients have usually suffered more severe TBI and polytrauma, reported both more severe depression and anxiety. These findings, together with inconsistent previous evidence [33], encourage further investigations to deepen our understanding concerning the neuropathology of mood disorders after TBI. For example, recent studies have identified changes in the white-matter microstructure and functional connectivity, providing support for a neurobiological basis of post-TBI mood/depressive disorders in more severe TBI [67,68].

The differential impact of depression and anxiety may pose important challenges to rehabilitation and recovery after TBI, if these mental problems are not diagnosed and treated early on during the clinical pathway. Additionally, also other psychological problems such as posttraumatic stress disorder may hamper recovery, which needs to be investigated in future research. Furthermore, it is of utter importance to also include the assessment of severity of TBI, as it is associated with depression combined with MEIs as they are associated with depression and anxiety and may therefore influence the recovery of individuals after TBI negatively.

This study has several strengths. Conducted using a large sample of the individuals after TBI in Europe and Israel, this study provides more stable results than studies with small samples. In addition, the study not only investigated all TBI severity groups, which allows TBI-severity-dependent conclusions to be drawn, but also includes a MEI assessment, enabling a differentiation of MD and GAD caused by head and non-head injuries.

A number of limitations need to be considered when interpreting the findings. First, although in line with current response rates in TBI research [15], participants in the current study were unevenly distributed with respect to the three clinical pathways. Due to the study design, outcome data were only collected up until six months post-injury among ER patients. Therefore, findings during the period of six to twelve months after TBI may not reflect the true effect among those from ERs and should be interpreted with caution. Second, the PHQ-9 and GAD-7 used for screening MD and GAD symptoms are both PROMs. Moreover, the reliance on a single source of information may introduce a rater bias [69] and the use of PROMs may inflate symptom reporting [9,10]. Therefore, future studies might benefit from consolidating self-reported screening instruments with standardized diagnostic interviews led by clinicians, such as the Mini International Neuropsychiatric Interview (MINI) [70] or the Structured Clinical Interview for DSM-5 disorders [71]. Also, somatic symptoms related to functional recovery status, for example, post-injury pain and fatigue, could potentially have an influence. Yet, they were not included in the current study due to the lack of valid corresponding data. Future studies should consider these factors. Furthermore, to avoid protentional bias, we excluded those individuals who reported having psychiatric problems prior to the TBI. Additional investigations focussing on this group and the reciprocity of anxiety and depression after TBI could not be performed due to the number of observations being too small for constructing latent factors and applying the cross-lagged analyses. However, the issue of anxiety and depression in this vulnerable group after TBI needs to be investigated in future studies. Additionally, a high proportion of missing information concerning the psychological services provided after TBI may limit drawing reliable conclusions about rehabilitation after hospital discharge. Finally, data were only available on the patients’ MD and GAD for the first year post-TBI. Future studies are warranted which examine this relationship over a longer period of time to deepen the understanding of the extent and complexity of factors that may modulate the development and trajectories of MD and GAD after TBI. In addition, studies are required to provide insight into the mechanisms of the association between depression, anxiety, functional recovery and rehabilitation outcomes after TBI, for which our study was not designed.

## 5. Conclusions

Based on a large European collaborative longitudinal study of patients with a clinical diagnosis of TBI, our findings provide new insights into the understanding of the longitudinal development and interplay of MD and GAD over the first year after TBI. Our results indicate that patient-reported MD and GAD are highly persistent and correlated after TBI. The longitudinal relationship between MD and GAD is reciprocal, with positive bi-directional impacts on each other. Being female, being admitted to an ICU, and being more severely disabled were risk factors associated with suffering from more severe MD and GAD. Injury-related factors were positively linked with MD and GAD after TBI. More specifically, MEI and major trauma was associated with both, whereas severity of TBI was only associated with MD. Our findings strongly suggest that early screening for and treatment of MD and GAD should be conducted in TBI patients to support rehabilitation within the first year post-injury.

## Figures and Tables

**Table 1:** Demographic characteristics of the effective sample (N = 1683).

	Number/Mean Score	Percentage/Standard Deviation
Female	563	33.5%
Age ^1^	49.25	19.55
Age group		
Adolescent (16–24)	263	15.6%
Young adult (25–34)	198	11.8%
Adult (35–44)	220	13.1%
Middle age (45–54)	280	16.6%
Upper middle age (55–64)	291	17.3%
Senior (≥65)	431	25.6%
Years of education ^1^	13.95	4.15
Education level		
None	13	0.9%
Currently studying	45	2.9%
Primary school	187	12.2%
Secondary/high school	529	34.6%
Post high school training	309	20.2%
College/university	446	29.2%
Employment status		
Employed, full-time	739	46.2%
Employed, part-time	132	11.4%
Sick leave	6	0.4%
Unemployed	91	5.7%
Retired	399	24.9%
Student	166	10.4%
Homemaker	17	1.1%
Relationship status		
Never been married	481	29.7%
Married	762	47.1%
Living together	152	9.4%
Divorced	115	7.1%
Separated	29	1.8%
Widowed	78	4.8%
Clinical pathway		
ADM	679	40.3%
ER	319	19.0%
ICU	685	40.7%
GCS score ^1^	12.85	3.74
GCS category		
Mild	1252	76.6%
Moderate	123	7.5%
Severe	259	15.9%
MEI	510	30.3%
Major trauma	838	50.1%
GOSE score ^1,2^	6.77	1.42
GOSE category		
Good recovery	1114	66.2%
Moderate disability	415	24.7%
Severe disability	154	9.2%

Notes: missing values n_employment_ = 82, n_relationship_ = 66, n_education years_ =280, n_education types_ = 154, n_GCS score/category_ = 49, n_major trauma_ = 9. TBI = Traumatic Brain Injury; ADM = admission to a hospital ward; ER = Emergency Room; ICU = Intensive Care Unit; GCS = Glasgow Coma Scale; MEI = Major Extracranial Injury; and GOSE = Glasgow Outcome Scale, Extended. ^1^ Instead of numbers and percentages, mean scores and standard deviations are reported. ^2^ All other demographic information was collected at baseline, except for the GOSE which was collected at six months after TBI.

**Table 2:** Descriptive statistics and correlations between the PHQ-9 and the GAD-7 at the scale level.

						Prevalence *			
	N	Cronbach’s α	Mean	SD	Min–Max [Q1, Q2, Q3]	Mild	Moderate	Moderately Severe	Severe
(1) PHQ-9 at 3 m	1519	0.86	4.71	4.89	0–25 [1, 3, 7]	24.9%	9.7%	4.5%	1.30%
(2) PHQ-9 at 6 m	1600	0.87	4.41	4.85	0–27 [1, 3, 6]	22.9%	8.4%	3.9%	1.80%
(3) PHQ-9 at 12 m	1156	0.87	4.46	4.98	0–27 [1, 3, 7]	20.4%	10.5%	3.2%	1.80%
(4) GAD-7 at 3 m	1514	0.90	3.29	4.20	0–21 [0, 2, 5]	18.0%	6.4%	-	3.10%
(5) GAD-7 at 6 m	1601	0.90	3.14	4.10	0–21 [0, 2, 5]	18.2%	5.2%	-	2.70%
(6) GAD-7 at 12 m	1160	0.90	3.11	4.03	0–21 [0, 2, 5]	17.8%	5.5%	-	2.50%

Notes: PHQ-9 = Patient Health Questionnaire-9; GAD-7 = General Anxiety Disorder-7; 3/6/12 m = 3/6/12-month post-TBI; Q1 = first quartile, 25%; Q2 = second quartile or median, 50%; Q3 = third quartile, 75%; * Cut-offs for different levels of major depression (MD) based on PHQ-9 scores were: 5–9 for mild MD, 10–14 for moderate MD, 15–19 for moderately severe MD, and above 20 for severe MD; and Cut-offs for different levels of generalized anxiety disorder (GAD) based on GAD-7 scores were: 5-9 for mild GAD, 10-14 for moderate GAD, and above 15 for severe GAD.

**Table 3:** Test of longitudinal measurement invariance of the latent constructs of depression and anxiety.

	Major Depression Assessed with the PHQ-9						Generalized Anxiety Disorder Assessed with the GAD-7					
Model	*χ2*	*df*	*p*-Value	CFI	TLI	RMSEA [90%CI]	*χ2*	*df*	*p*-Value	CFI	TLI	RMSEA [90%CI]
Baseline model	980.700	294	<0.001	0.982	0.979	0.037 [0.035–0.040]	635.441	165	<0.001	0.990	0.987	0.041 [0.038–0.045]
Loading invariance	998.087	310	<0.001	0.982	0.980	0.036 [0.034–0.039]	665.040	177	<0.001	0.989	0.987	0.040 [0.037–0.044]
Threshold invariance	1004.896	344	<0.001	0.983	0.983	0.034 [0.031–0.036]	675.662	203	<0.001	0.990	0.989	0.037 [0.034–0.040]
Unique factor invariance	910.185	362	<0.001	0.986	0.986	0.030 [0.028–0.032]	621.769	217	<0.001	0.991	0.991	0.033 [0.030–0.036]

Notes: PHQ-9 = Patient Health Questionnaire-9; GAD-7 = General Anxiety Disorder-7; CFI = comparative fit index; TLI = Tucker–Lewis index; RMSEA = root mean square error of approximation; and 90% CI = confidence interval (lower bound and upper bound in square brackets).

**Table 4:** Model selection criteria to determine the best fitting model of the reciprocal relations between MD and GAD after TBI.

Model		*χ2*	*df*	*p*-Value	CFI	TLI	RMSEA [90%CI]
**1**	autoregressive paths between adjacent time points	2522.981	1025	<0.001	0.982	0.980	0.029 [0.028–0.031]
**2**	add cross-lagged paths between adjacent time points	2523.358	1021	<0.001	0.982	0.980	0.030 [0.028–0.031]
**3**	**add autoregressive paths between distant time points**	**2338.889**	**1019**	**<0.001**	**0.984**	**0.983**	**0.028 [0.026–0.029]**
**4**	add cross-lagged paths between distant time points	2347.931	1017	<0.001	0.984	0.983	0.028 [0.026–0.029]

Notes: Models 1 through 4 were nested within each other. CFI = comparative fit index; TLI = Tucker–Lewis index; RMSEA = root mean square error of approximation; and 90% CI = confidence interval (lower bound and upper bound in square brackets). The final model is shown in **bold** font.

**Table 5:** Risk factors for higher levels of depression and anxiety after TBI.

		Depression		Anxiety	
Covariate ^1^	Reference	*β* ^3^	*p*-Value ^4^	*β* ^3^	*p*-Value ^4^
Sex	Male	0.211	**<0.001**	0.207	**<0.001**
Age	-	−0.068	0.020	−0.088	**0.0030**
Years of education	-	−0.057	0.086	−0.046	0.158
Employment status	Employed	−0.047	0.44	−0.102	0.098
Relationship status	Stable relationship	0.172	**0.0042**	0.106	0.080
Clinical pathway					
ER	Adm	0.051	0.047	0.006	0.85
ICU	Adm	0.333	**<0.001**	0.222	**<0.001**
GCS score	-	−0.110	**<0.001**	−0.043	0.15
MEI	No	0.244	**<0.001**	0.231	**<0.001**
Major trauma	No	0.219	**<0.001**	0.154	0.0093
GOSE score at 6m after TBI ^2^	-	−0.443	**<0.0** **0** **01**	−0.337	**<0.001**
	-	−0.216	**<0.001**	−0.142	**<0.001**

Notes: TBI = Traumatic Brain Injury; ADM = admission to a hospital ward; ER = Emergency Room; ICU = Intensive Care Unit; GCS = Glasgow Coma Scale; MEI = Major Extracranial Injury; and GOSE = Glasgow Outcome Scale, Extended. ^1^ Age, years of education, number of psychiatric problems prior to TBI, GCS and GOSE were used as continuous covariates. All other variables were used as nominal covariates with a reference category. All variables were measured at baseline, except the GOSE score at six months after TBI. ^2^ The first row shows depression and anxiety at six months after TBI, the second row indicates depression and anxiety at twelve months after TBI. ^3^ The *β* coefficient of continuous covariates represents how many units/standard deviations of change in depression or anxiety will occur per unit/standard deviation of change in the continuous covariate. The *β* coefficient of the nominal covariates represents how many units/standard deviations of change in depression or anxiety will be observed when compared with the reference category. ^4^ *p* values in **bold** are significant after Bonferroni correction with the significance threshold at 0.050/12 = 0.0042.

## Data Availability

All relevant data are available upon request from CENTER-TBI, and the authors are not legally allowed to share it publicly. The authors confirm that they received no special access privileges to the data. CENTER-TBI is committed to data sharing and in particular to responsible further use of the data. Hereto, we have a data sharing statement in place: https://www.center-tbi.eu/data/sharing, accessed on 4 November 2021. The CENTER-TBI Management Committee, in collaboration with the General Assembly, established the Data Sharing policy, and Publication and Authorship Guidelines to assure correct and appropriate use of the data as the dataset is hugely complex and requires help of experts from the Data Curation Team or Bio- Statistical Team for correct use. This means that we encourage researchers to contact the CENTER-TBI team for any research plans and the Data Curation Team for any help in appropriate use of the data, including sharing of scripts. Requests for data access can be submitted online: https://www.center-tbi.eu/data, accessed on 4 November 2021. The complete Manual for data access is also available online: https://www.center-tbi.eu/files/SOP-Manual-DAPR-20181101.pdf, accessed on 4 November 2021.

## Supplementary Materials

The following materials are available online at https://www.mdpi.com/article/10.3390/jcm10235597/s1, Supplemental Notes 1: Measures of Patient Characteristics (1.1. Sociodemographic factors, 1.2. Injury-related factors), Supplemental Notes 2: Statistical Analysis (2.1. Descriptive statistics and item characteristics, 2.2. Longitudinal measurement invariance, 2.3. Autoregressive cross-lagged models, 2.4. Risk factors), Supplemental Notes 3: Results (3.1. Longitudinal measurement invariance, 3.2. Model selection). Supplemental Table S1: Categorical variables of selected and unselected individuals, Supplemental Table S2: Continuous variables of selected and unselected individuals, Supplemental Table S3: Unselected individuals with psychiatric/psychological problems prior to TBI, Supplemental Table S4: Participants with valid measurements of depression and anxiety after TBI per clinical pathway, Supplemental Table S5: Descriptive statistics of detailed symptoms of depression and anxiety after TBI, Supplemental Table S6: Moderate to severe rate of depression and anxiety according to TBI with and without MEI, Supplemental Table S7: Psychological treatment at 3, 6 and 12 months during rehabilitation after TBI, Supplemental Table S8: Latent structure of depression and anxiety after TBI. Appendix 1: The CENTER-TBI participants and investigators, Appendix 2: STROBE Statement, Supplemental References.
