# Supplementary material for: Longitudinal Analyses of the Reciprocity of Depression and Anxiety after Traumatic Brain Injury and Its Clinical Implications
Source: J Clin Med. 2021 Nov 28;10(23):5597. doi: 10.3390/jcm10235597 (PMC8658198; doi:10.3390/jcm10235597)
Supplement: Supplementary file 1 [file jcm-10-05597-s001.zip › jcm-1472985-supplementary.pdf]

SUPPLEMENTARY MATERIAL

Longitudinal Analyses of the Reciprocity of Depression and Anxiety after Traumatic Brain Injury and Its Clinical Implications

Biyao Wang [1,2,*], Marina Zeldovich [1], Katrin Rauen [3,4], Yi-Jhen Wu [1], Amra Covic, Isabelle Müller [1], Juanita Haagsma [5,6], Suzanne Polinder [5], David Menon [7], Thomas Asendorf [8], Nada Andelic [9,10], Nicole v. Steinbüchel [1], and CENTER-TBI participants and investigators[†]

[1] Institute of Medical Psychology and Medical Sociology, University Medical Center Goettingen, Waldweg 37A, 37073 Goettingen, Germany; marina.zeldovich@med.uni-goettingen.de (M.Z.); yi-jhen.wu@med.uni-goettingen.de (Y.-J.W.); isabelle.mueller@med.uni-goettingen.de (I.M.); nvsteinbuechel@med.uni-goettingen.de (N.v.S.)
[2] Division of Psychology and Language Sciences, University College London, WC1H 0AP London, UK
[3] Department of Geriatric Psychiatry, Psychiatric Hospital Zurich, University of Zurich, Minervastrasse 145, 8032 Zurich, Switzerland; katrin.rauen@uzh.ch
[4] Institute for Stroke and Dementia Research (ISD), University Hospital, LMU Munich, Feodor-Lynen-Straße 17, 81377 Munich, Germany
[5] Department of Public Health, Erasmus MC, University Medical Center Rotterdam, 3000 CA Rotterdam, The Netherlands; j.haagsma@erasmusmc.nl (J.H.); s.polinder@erasmusmc.nl (S.P.)
[6] Department of Emergency Medicine, Erasmus MC, University Medical Center Rotterdam, 3000 CA Rotterdam, The Netherlands
[7] Division of Anaesthesia, University of Cambridge/Addenbrooke's Hospital, Box 157, Cambridge CB2 0QQ, UK; dkm13@cam.ac.uk
[8] Department of Medical Statistics, University Medical Center Goettingen, 37073 Goettingen, Germany; thomas.asendorf@med.uni-goettingen.de
[9] Department of Physical Medicine and Rehabilitation, Oslo University Hospital, 0424 Oslo, Norway; NADAND@ous-hf.no
[10] Research Centre for Habilitation and Rehabilitation Models and Services (CHARM), Faculty of Medicine, Institute of Health and Society, University of Oslo, 0373 Oslo, Norway
* Correspondence: Biyao.Wang@med.uni-goettingen.de
† The full list of the CENTER-TBI participants and investigators is provided in the Online Supplement

Supplemental Notes

1. Measures of Patient Characteristics

1.1. Sociodemographic factors

Socio-demographic information was collected at the time of enrolment into the study (baseline), including the participants' sex, age, employment status, relationship status, and education level.

1.2. Injury-related factors

The patient's clinical pathway was collected, including admission to a hospital ward, the emergency room and the intensive care unit. The Glasgow Coma Scale (GCS) was used to assess the severity of TBI and the level of consciousness after TBI [1]. The GCS classifies patients' impairment of consciousness from 15 (fully conscious) to 3

(brain death or those in deep unconsciousness). The GCS was calculated centrally and implemented in the database with missing values imputed using the IMPACT methodology [2]. In the current study, we refer to GCS scores of 13 -15 as mild injury, 9-12 as moderate injury, and 3-8 as severe injury. The Abbreviated Injury Scale (AIS) is an anatomical, consensus-derived, global severity scoring system that classifies each injury by body region [3]. Injury was rated on a 6-point ordinal scale (1 = minor, 2 = moderate, 3 = serious, 4 = severe, 5 = critical, and 6 = unsurvivable). Major Extracranial Injury (MEI) was defined as an AIS ≥3 in any non-head and neck region[4]. By taking the sum of squares of the highest AIS severity score in each of the three most severely injured body regions, the Injury Severity Score (ISS) can be calculated. Major trauma was defined as an ISS ≥ 16[5, 6]. The Glasgow Outcome Scale, Extended (GOSE) was used to assess functional disabilities and recovery after TBI [7]. Functional outcomes were clinician-rated and included eight states: dead (1), vegetative state (2), lower severe disability (3), upper severe disability (4), lower moderate disability (5), upper moderate disability (6), lower good recovery (7) and upper good recovery (8). In addition, the self- or proxy-reported version of the GOSE questionnaire (GOSE-Q)[8] was used to collect the information on functional status after TBI by regular mail. The GOSE-Q score was centrally computed and combined with the GOSE interview score in the core database to optimise data availability. As the GOSE-Q cannot distinguish between a vegetative state (2) and the lower severe disability (3) of those affected, both categories were collapsed into one group (2/3). Individuals included in this study had a GOSE score equal to or higher than 2/3. We refer to individuals with GOSE states 7-8 as good recovery, 5-6 as moderate disability, less or equal to 4 as severe disability [7].

2. Statistical Analysis

2.1. Descriptive statistics and item characteristics

Symptoms of depression and anxiety measured using the PHQ-9 and GAD-7 and potential risk factors in the effective sample were reported descriptively. The comparison between individuals included and the ones excluded from the analyses was reported. The internal consistencies of the PHQ-9 and GAD-7 were analysed separately using Cronbach's alpha and Pearson correlations between symptoms of depression and anxiety and are reported separately for each time point.

2.2. Longitudinal measurement invariance

The psychological constructs of depression and anxiety were used as unobserved latent variables under the Structural Equation Modelling (SEM) framework [9], measured using observed symptoms (nine symptoms for depression, seven symptoms for anxiety). Therefore, longitudinal measurement invariance (MI) [10] of each latent construct was a prerequisite for the following analytical steps, to ensure that depression and anxiety assessed at each measurement point represent the same latent profile, i.e., that any observed changes over time reflected true changes in the level of depression or anxiety, rather than changes in the latent structure. Since

symptoms of depression and anxiety were measured as categorical variables, we followed the procedure recommended by Liu [11]. The longitudinal MI testing consisted of a sequence of nested models with increasingly restrictive model constraints. (1) Configural invariance model is the baseline model, testing the hypothesis that the same factor structure (i.e., general pattern of factor loadings) holds across time. This baseline model should provide a good fit to the data in order to continue the evaluation of weak to strict invariance models. (2) The loading invariance model represents the weak invariance model and adds the constraint that factor loadings are identical across time. (3) The threshold invariance model is the strong invariance model. It adds the constraint that for each indicator the threshold level of changing from one response category to the next is identical over time. (4) The unique factor invariance model represents the strict invariance model and adds the constraint that the unique factor variances are equal over time. For the comparison between two nested models (i.e., configural vs. weak, weak vs. strong, strong vs. strict), a change of ≥ .010 in the comparative fit indices (CFI) [12], supplemented by a change of ≥ .015 in the root mean square of approximation (RMSEA) [13] would indicate non-invariance [14].

2.3. Autoregressive cross-lagged models

To identify the reciprocal longitudinal relationships between depression and anxiety after TBI, we applied autoregressive cross-lagged (ARCL) models, which allow longitudinal influences between constructs to be explored while controlling for their concurrent associations and the stability within each construct over time [15-17]. Here, autoregressive (AR) effects describe the effect of a construct on itself measured at a later time point, with larger AR coefficients indicating lower inter-individual variance from the previous time point, i.e. greater stability [18]. Cross-lagged (CL) effects describe the influence that one construct has on another measured at a later time point, after controlling for their concurrent and AR effects. With depression and anxiety 3, 6, and 12 months after TBI used as outcome variables, AR and CL models were built in a cumulative way: the first model was the base model in which only AR paths between adjacent time points (i.e., 3 months vs. 6 months and 6 months vs. 12 months) were included; the second model added CL paths between adjacent time points on this basis; the third model included AR paths between distant time points (i.e., 3 months vs. 12 months); the fourth model further included CL paths between distant time points, which made it the full model including all possible paths. Those models were nested with each other and compared with multiple model fit criteria. The chi-square (χ^2) index was reported, with a non-significant chi-square reflecting a good fit to the data. However, chi-square is sensitive to sample size and may reflect statistical significance even though a model fits the data well [19, 20]. Other model selection criteria included CFI, RMSEA, the Tucker-Lewis index (TLI) [21]. As recommended, TLI and CFI value >.95, RMSEA values <.05, respectively, indicate a good fit with the data [22].

2.4. Risk factors

Potential risk factors were separately added to the optimal ARCL model as covariates to investigate how they are associated with the level of depression and anxiety. Age, education level (years of education), functional disabilities and recovery (GOSE score), severity of TBI (GCS score) were considered as continuous variables and used directly in the model, while sex, clinical pathway, employment status, relationship status, major extracranial injury and major trauma were treated as nominal variables and transformed into dummy variables. Categories were recoded as follows: employed full-time or part-time and sick leave were merged to employed; retired, student, homemaker and unemployed were merged to unemployed; married and living together were merged to in a stable relationship; divorced, separated and widowed were merged to not in a stable relationship. The reference group for categorical variables was female, from ADM, in a relationship, unemployed, no major extracranial injury and no major trauma. All covariates were measured at baseline, except for the GOSE score. Here, we used the GOSE score assessed at six months to examine the association between functional disability and recovery after TBI and emotional status.

3. Results

3.1. Longitudinal measurement invariance

According to the main model, goodness of fit indexes (CFI and RMSEA), for both depression and anxiety, displayed a good and equivalent fit to data in all the above-mentioned models (Table 3), which indicates that the latent profile of depression and anxiety measured at each time point represented the same underlying construct and could therefore be used across time points in further longitudinal modelling. Standardised factor loadings for the latent constructs of depression and anxiety were high across time points (Table S8).

3.2. Model selection

To build the AR and CL model which best represents the longitudinal reciprocal relationship between depression and anxiety after TBI, several nested models were compared in Table 4. The baseline model (Model 1) only included AR pathways between adjacent time points, adding CL pathways between adjacent time points (Model 2) and AR pathways between distant time points (Model 3) improved the model fit to data. However, further including CL pathways between distant time points (Model 4) did not enhance the model fit, nor did it reveal any additional significant pathways. Model 3 was therefore chosen as the optimal model.

Supplemental Tables

Table S1. Categorical variables of selected and unselected individuals

Variable	Selected		Not selected		Chi-square test[2]		
	n=1683	%	n=928	%	χ^2	*df*	*p*-value[3]
Sex					1.891	1	0.17
Male	1250	65.0%	592	63.8%			
Female	563	33.5%	336	36.2%			
Age group					6.765	5	0.24
16-24	283	14.7%	144	15.5%			
25-34	227	11.8%	137	14.8%			
35-44	256	13.3%	119	12.8%			
45-54	330	17.2%	139	15.0%			
55-64	348	18.1%	171	18.4%			
>=65	478	24.9%	218	23.5%			
Employment status					41.336	6	**<0.001**
Employed, full-time	825	45.5%	349	41.3%			
Employed, part-time	209	11.5%	77	9.1%			
Sick leave	11	0.6%	10	1.2%			
Unemployed	124	6.8%	97	11.5%			
Retired	447	24.6%	215	25.4%			
Student	177	9.8%	79	9.3%			
Homemaker	21	1.2%	19	2.2%			
Relationship status					19.888	5	0.0013
Never been married	546	29.7%	287	33.9%			
Married	840	45.8%	336	39.7%			
Living together	173	9.4%	70	8.3%			
Divorced	138	7.5%	68	8.0%			
Separated	43	2.3%	25	3.0%			
Widowed	96	5.2%	60	7.1%			
Education level					12.880	5	0.025
None	16	0.9%	15	1.9%			
Currently studying	48	2.8%	12	2.8%			
Primary school	215	12.4%	116	14.7%			
Secondary/high school	597	34.4%	274	34.7%			
Post high school training	357	20.6%	173	21.9%			
College/university	503	29.0%	190	24.1%			
Clinical pathway					12.715	2	**0.0017**
ADM	761	39.6%	315	33.9%			
ER	367	19.1%	217	23.4%			
ICU	794	41.3%	396	42.7%			
GCS					2.726	2	0.26
Mild	1425	76.3%	661	73.9%			
Moderate	136	7.3%	69	7.7%			
Severe	306	16.4%	164	18.3%			
MEI					0.514	1	0.47
No	1342	69.8%	660	71.1%			

Yes	580	30.2%	268	28.9%			
Major trauma					0.063	1	0.80
No	953	50.0%	446	49.3%			
Yes	953	50.0%	458	50.7%			
GOSE at 6m after TBI[1]					31.801	2	**< 0.001**
Good recovery	1235	64.3%	531	57.9%			
Moderate disability	512	26.6%	238	26.0%			
Severe disability	175	9.1%	148	16.1%			

Notes. TBI = Traumatic Brain Injury; ER = Emergency Room; ADM = admission to a hospital ward; ICU = Intensive Care Unit; GCS = The Glasgow Coma Scale; MEI = Major Extracranial Injury; GOSE = The Glasgow Outcome Scale - Extended.

[1] All other demographic information was measured at baseline.

[2] Employment and relationship status were recoded according to the categories described in the statistical analyses section before testing.

[3] *p* values in bold are significant after Bonferroni correction with the significance threshold at 0.050/10 = 0.005.

Table S2. Continuous variables of selected and unselected individuals

Variable	Selected			Not selected			t-test		
	N	Mean	SD	N	Mean	SD	t	df	p-value[1]
Age	1922	49.44	19.250	928	48.41	19.45	-1.053	2609	0.29
Years of education				719	12.92	3.84	-5.515	2120	**<0.001**
GCS score				920	12.59	3.94	-1.646	2526	0.10
GOSE score				894	6.38	1.71	-6.316	2601	**<0.001**
PHQ-9 (3-month post-TBI)	1729	5.19	5.209	523	7.07	6.53	8.684	2040	**<0.001**
PHQ-9 (6-month post-TBI)	1831	4.84	5.134	522	7.05	6.21	10.050	2120	**<0.001**
PHQ-9 (12-month post-TBI)	1310	4.92	5.334	252	7.29	6.44	7.749	1406	**<0.001**
GAD-7 (3-month post-TBI)	1724	3.64	4.471	522	4.98	5.37	7.389	2034	**<0.001**
GAD-7 (6-month post-TBI)	1832	3.44	4.351	519	5.17	5.44	9.004	2118	**<0.001**
GAD-7 (12-month post-TBI)	1315	3.43	4.311	256	5.22	5.37	7.111	1414	**<0.001**

Notes: PHQ-9=The Patient Health Questionnaire-9; GAD-7 = The General Anxiety Disorder-7; TBI = Traumatic Brain Injury; GCS = The Glasgow Coma Scale; GOSE = The Glasgow Outcome Scale, Extended.

[1] *p* values in bold are significant after Bonferroni correction with the significance threshold at 0.050/10 = 0.005.

Table S3. Unselected individuals with psychiatric/psychological problems prior to TBI

	Number	Percentage
Prior psychiatric problem		
Any prior psychiatric problem	318	34.3%
Prior depression	181	19.5%
Prior anxiety	80	8.6%
Prior sleep disorders	50	5.4%
Prior schizophrenia	6	0.6%
Prior substance use	64	6.9%
Other prior problem	57	6.1%
Number of psychiatric problems		
0	610	65.7%
1	235	25.3%
2	54	5.8%
3	23	2.5%
4	4	0.4%
5	2	0.2%

Notes: unselected individuals N = 928. TBI = Traumatic Brain Injury

Table S4. Participants with valid measurements of depression and anxiety after TBI per clinical pathway

	Valid measurement of PHQ-9						Valid measurement of GAD-7					
	Valid time point			Valid n of measurements			Valid time point			Valid n of measurements		
	3m	6m	12m	n=1	n=2	n=3	3m	6m	12m	n=1	n=2	n=3
ADM	616	632	564	229	260	505	614	632	565	231	259	504
ER	319	319	0	165	371	0	319	319	0	167	369	0
ICU	584	649	592	282	278	521	581	650	595	279	279	523
Total	1519	1600	1156	676	909	1026	1514	1601	1160	677	907	1027

Notes. PHQ-9 = The Patient Health Questionnaire-9; GAD-7 = The General Anxiety Disorder-7; ER = Emergency Room; ADM = admission to a hospital ward; ICU = Intensive Care Unit; Time point = participant with a valid measurement at a certain assessment time point; 3m = 3-month post-TBI; 6m = 6-month post-TBI; 12m = 12-month post-TBI; valid assessments = participant who has 1, 2, or 3 valid measurements at 3, 6, 12 months after TBI.

Table S5. Descriptive statistics of detailed symptoms of depression and anxiety after TBI

		3-month post-TBI			6-month post-TBI			12-month post-TBI		
		N	Mean	SD	N	Mean	SD	N	Mean	SD
PHQ-9f	Little interest	N.3m	M.3m	SD.3m	N.6m	M.6m	SD.6m	N.12m	M.12m	SD.12m
	Feeling down	1518	0.543	0.543	1600	0.531	0.531	1156	0.499	0.499
	Trouble sleeping	1517	0.503	0.503	1601	0.467	0.467	1156	0.458	0.458
	Feeling tired	1519	0.854	0.854	1600	0.765	0.765	1157	0.787	0.787
	Poor appetite	1519	1.011	1.011	1600	0.937	0.937	1157	0.935	0.935
	Low self-esteem	1518	0.482	0.482	1600	0.456	0.456	1157	0.44	0.44
	Trouble concentrating	1519	0.357	0.357	1599	0.342	0.342	1156	0.373	0.373
	Moving slowly	1520	0.555	0.555	1599	0.504	0.504	1156	0.54	0.54
	Thoughts of death	1519	0.28	0.28	1600	0.258	0.258	1156	0.265	0.265
GAD-7	Feeling nervous	1518	0.132	0.132	1598	0.146	0.146	1155	0.155	0.155
	Non-stop worrying	1514	0.53	0.53	1601	0.515	0.515	1159	0.5	0.5
	Worry too much	1512	0.475	0.475	1600	0.451	0.451	1160	0.415	0.415
	Trouble relaxing	1513	0.59	0.59	1599	0.548	0.548	1157	0.529	0.529
	Restless	1514	0.54	0.54	1600	0.492	0.492	1158	0.519	0.519
	Easily annoyed	1514	0.334	0.334	1600	0.299	0.299	1160	0.297	0.297
	Feeling afraid	1514	0.515	0.515	1600	0.536	0.536	1160	0.558	0.558

Notes: PHQ-9=The Patient Health Questionnaire-9; GAD-7 = The General Anxiety Disorder-7; TBI = Traumatic Brain Injury.

Table S6. Moderate to severe rate of depression and anxiety according to TBI with and without MEI

	Isolate TBI		TBI with MEI		Chi-square test		
	N	Moderate/ severe rate[1]	N	Moderate/ severe rate[1]	χ^2	*df*	*p*-value[2]
PHQ-9 at 3m	1072	14.3%	447	18.6%	4.12	1	0.043
PHQ-9 at 6m	1122	13.0%	478	16.5%	3.14	1	0.076
PHQ-9 at 12m	723	14.2%	433	17.6%	2.02	1	0.16
GAD-7 at 3m	1068	8.8%	446	11.2%	1.85	1	0.17
GAD-7 at 6m	1121	7.6%	480	8.8%	0.48	1	0.49
GAD-7 at 12m	727	7.3%	433	9.2%	1.14	1	0.28

Notes: MEI = Major Extracranial Injury; PHQ-9 = Patient Health Questionnaire-9; GAD-7 = General Anxiety Disorder-7; 3/6/12m = 3/6/12-month post-TBI

[1] based on the cut-off = 10

[2] *p* values in bold are significant after Bonferroni correction with the significance threshold at 0.050/6 = 0.0083.

Table S7. Psychological treatment at 3, 6 and 12 months during rehabilitation after TBI.

	Total sample n=386[2]				Over PHQ-9 cut-off[1] n=148[2]				Over GAD-7 cut-off[1] n=77[2]			
	Yes		No		Yes		No		Yes		No	
	n	%	n	%	n	%	n	%	n	%	n	%
Treatment at 3-month post-TBI	32	26.4%	89	73.6%	18	32.1%	38	67.9%	8	26.7%	22	73.3%
Treatment at 6-month post-TBI	27	14.5%	159	85.5%	15	22.7%	51	77.3%	10	30.3%	23	69.7%
Treatment at 12-month post-TBI	51	21.4%	187	78.6%	30	30.3%	69	69.7%	19	36.5%	33	63.5%
Ever received treatment during rehabilitation	84	21.8%	302	78.2%	50	33.8%	98	66.2%	29	37.7%	48	62.3%

Notes: PHQ-9 = The Patient Health Questionnaire-9; GAD-7 = The General Anxiety Disorder-7; TBI = Traumatic Brain Injury.

[1] the sample of patients who had depression or anxiety at any measurement point.

[2] Information on psychological treatment was only available for a small proportion of TBI patients, therefore the rate of yes (received treatment) and no (did not receive treatment) is calculated based on the available data. Missing values n_{total} = 1297, n_{everMD} = 244, $n_{everGAD}$ = 160.

Table S8. Latent structure of depression and anxiety after TBI

A. Latent construct of depression as indicated by items from PHQ-9

	3-month post-TBI	6-month post-TBI	12-month post-TBI
Little interest	0.836	0.820	0.829
Feeling down	0.898	0.901	0.907
Trouble sleeping	0.667	0.642	0.658
Feeling tired	0.752	0.762	0.790
Poor appetite	0.710	0.684	0.683
Low self-esteem	0.798	0.825	0.838
Trouble concentrating	0.752	0.767	0.756
Moving slowly	0.692	0.724	0.749
Thoughts of death	0.727	0.773	0.785

B. Latent construct of anxiety as indicated by items from the GAD-7

	3-month post-TBI	6-month post-TBI	12-month post-TBI
Feeling nervous	0.872	0.868	0.869
Non-stop worrying	0.930	0.925	0.923
Worry too much	0.873	0.890	0.897
Trouble relaxing	0.872	0.885	0.890
Restless	0.776	0.786	0.809
Easily annoyed	0.767	0.798	0.792
Feeling afraid	0.780	0.767	0.786

Notes: PHQ-9=The Patient Health Questionnaire-9; GAD-7 = The General Anxiety Disorder-7; TBI = Traumatic Brain Injury. Latent structure of depression and anxiety are shown as standardised factor loadings of corresponding items from the PHQ-9 and the GAD-7

All factor loadings were significant at the $p < .001$ level.

Appendix

Appendix 1: The CENTER-TBI participants and investigators

Cecilia Åkerlund[1], Krisztina Amrein[2], Nada Andelic[3], Lasse Andreassen[4], Audny Anke[5], Anna Antoni[6], Gérard Audibert[7], Philippe Azouvi[8], Maria Luisa Azzolini[9], Ronald Bartels[10], Pál Barzó[11], Romuald Beauvais[12], Ronny Beer[13], Bo-Michael Bellander[14], Antonio Belli[15], Habib Benali[16], Maurizio Berardino[17], Luigi Beretta[9], Morten Blaabjerg[18], Peter Bragge[19], Alexandra Brazinova[20], Vibeke Brinck[21], Joanne Brooker[22], Camilla Brorsson[23], Andras Buki[24], Monika Bullinger[25], Manuel Cabeleira[26], Alessio Caccioppola[27], Emiliana Calappi [27], Maria Rosa Calvi[9], Peter Cameron[28], Guillermo Carbayo Lozano[29], Marco Carbonara[27], Simona Cavallo[17], Giorgio Chevallard[30], Arturo Chieregato[30], Giuseppe Citerio[31, 32], Hans Clusmann[33], Mark Coburn[34], Jonathan Coles[35], Jamie D. Cooper[36], Marta Correia[37], Amra Čović[38], Nicola Curry[39], Endre Czeiter[24], Marek Czosnyka[26], Claire Dahyot-Fizelier[40], Paul Dark[41], Helen Dawes[42], Véronique De Keyser[43], Vincent Degos[16], Francesco Della Corte[44], Hugo den Boogert[10], Bart Depreitere[45], Đula Đilvesi[46], Abhishek Dixit[47], Emma Donoghue[22], Jens Dreier[48], Guy-Loup Dulière[49], Ari Ercole[47], Patrick Esser[42], Erzsébet Ezer[50], Martin Fabricius[51], Valery L. Feigin[52], Kelly Foks[53], Shirin Frisvold[54], Alex

Furmanov[55], Pablo Gagliardo[56], Damien Galanaud[16], Dashiell Gantner[28], Guoyi Gao[57], Pradeep George[58], Alexandre Ghuysen[59], Lelde Giga[60], Ben Glocker[61], Jagoš Golubovic[46], Pedro A. Gomez [62], Johannes Gratz[63], Benjamin Gravesteijn[64], Francesca Grossi[44], Russell L. Gruen[65], Deepak Gupta[66], Juanita A. Haagsma[64], Iain Haitsma[67], Raimund Helbok[13], Eirik Helseth[68], Lindsay Horton [69], Jilske Huijben[64], Peter J. Hutchinson[70], Bram Jacobs[71], Stefan Jankowski[72], Mike Jarrett[21], Ji-yao Jiang[58], Faye Johnson[73], Kelly Jones[52], Mladen Karan[46], Angelos G. Kolias[70], Erwin Kompanje[74], Daniel Kondziella[51], Evgenios Kornaropoulos[47], Lars-Owe Koskinen[75], Noémi Kovács[76], Ana Kowark[77], Alfonso Lagares[62], Linda Lanyon[58], Steven Laureys[78], Fiona Lecky[79, 80], Didier Ledoux[78], Rolf Lefering[81], Valerie Legrand[82], Aurelie Lejeune[83], Leon Levi[84], Roger Lightfoot[85], Hester Lingsma[64], Andrew I.R. Maas[43], Ana M. Castaño-León[62], Marc Maegele[86], Marek Majdan[20], Alex Manara[87], Geoffrey Manley[88], Costanza Martino[89], Hugues Maréchal[49], Julia Mattern[90], Catherine McMahon[91], Béla Melegh[92], David Menon[47], Tomas Menovsky[43], Ana Mikolic[64], Benoit Misset[78], Visakh Muraleedharan[58], Lynnette Murray[28], Ancuta Negru[93], David Nelson[1], Virginia Newcombe[47], Daan Nieboer[64], József Nyirádi[2], Otesile Olubukola[79], Matej Oresic[94], Fabrizio Ortolano[27], Aarno Palotie[95, 96, 97], Paul M. Parizel[98], Jean-François Payen[99], Natascha Perera[12], Vincent Perlbarg[16], Paolo Persona[100], Wilco Peul[101], Anna Piippo-Karjalainen[102], Matti Pirinen[95], Horia Ples[93], Suzanne Polinder[64], Inigo Pomposo[29], Jussi P. Posti [103], Louis Puybasset[104], Andreea Radoi [105], Arminas Ragauskas[106], Rahul Raj[102], Malinka Rambadagalla[107], Jonathan Rhodes[108], Sylvia Richardson[109], Sophie Richter[47], Samuli Ripatti[95], Saulius Rocka[106], Cecilie Roe[110], Olav Roise[111,112], Jonathan Rosand[113], Jeffrey V. Rosenfeld[114], Christina Rosenlund[115], Guy Rosenthal[55], Rolf Rossaint[77], Sandra Rossi[100], Daniel Rueckert[61] Martin Rusnák[116], Juan Sahuquillo[105], Oliver Sakowitz[90, 117], Renan Sanchez-Porras[117], Janos Sandor[118], Nadine Schäfer[81], Silke Schmidt[119], Herbert Schoechl[120], Guus Schoonman[121], Rico Frederik Schou[122], Elisabeth Schwendenwein[6], Charlie Sewalt[64], Toril Skandsen[123, 124], Peter Smielewski[26], Abayomi Sorinola[125], Emmanuel Stamatakis[47], Simon Stanworth[39], Robert Stevens[126], William Stewart[127], Ewout W. Steyerberg[64, 128], Nino Stocchetti[129], Nina Sundström[130], Riikka Takala[131], Viktória Tamás[125], Tomas Tamosuitis[132], Mark Steven Taylor[20], Braden Te Ao[52], Olli Tenovuo[103], Alice Theadom[52], Matt Thomas[87], Dick Tibboel[133], Marjolein Timmers[74], Christos Tolias[134], Tony Trapani[28], Cristina Maria Tudora[93], Andreas Unterberg[90], Peter Vajkoczy [135], Shirley Vallance[28], Egils Valeinis[60], Zoltán Vámos[50], Mathieu van der Jagt[136], Gregory Van der Steen[43], Joukje van der Naalt[71], Jeroen T.J.M. van Dijck [101], Thomas A. van Essen[101], Wim Van Hecke[137], Caroline van Heugten[138], Dominique Van Praag[139], Thijs Vande Vyvere[137], Roel P. J. van Wijk[101], Alessia Vargiolu[32], Emmanuel Vega[83], Kimberley Velt[64], Jan Verheyden[137], Paul M. Vespa[140], Anne Vik[123, 141], Rimantas Vilcinis[132], Victor Volovici[67], Nicole von Steinbüchel[38], Daphne Voormolen[64], Petar Vulekovic[46], Kevin K.W. Wang[142], Eveline Wiegers[64], Guy Williams[47], Lindsay Wilson[69], Stefan Winzeck[47], Stefan Wolf[143], Zhihui Yang[113], Peter Ylén[144], Alexander Younsi[90], Frederick A. Zeiler[47,145], Veronika Zelinkova[20], Agate Ziverte[60], Tommaso Zoerle[27]

[1] Department of Physiology and Pharmacology, Section of Perioperative Medicine and Intensive Care, Karolinska Institutet, Stockholm, Sweden
[2] János Szentágothai Research Centre, University of Pécs, Pécs, Hungary
[3] Division of Surgery and Clinical Neuroscience, Department of Physical Medicine and Rehabilitation, Oslo University Hospital and University of Oslo, Oslo, Norway
[4] Department of Neurosurgery, University Hospital Northern Norway, Tromso, Norway
[5] Department of Physical Medicine and Rehabilitation, University Hospital Northern Norway, Tromso, Norway
[6] Trauma Surgery, Medical University Vienna, Vienna, Austria
[7] Department of Anesthesiology & Intensive Care, University Hospital Nancy, Nancy, France
[8] Raymond Poincare hospital, Assistance Publique – Hopitaux de Paris, Paris, France
[9] Department of Anesthesiology & Intensive Care, S Raffaele University Hospital, Milan, Italy
[10] Department of Neurosurgery, Radboud University Medical Center, Nijmegen, The Netherlands
[11] Department of Neurosurgery, University of Szeged, Szeged, Hungary
[12] International Projects Management, ARTTIC, Munchen, Germany
[13] Department of Neurology, Neurological Intensive Care Unit, Medical University of Innsbruck, Innsbruck, Austria
[14] Department of Neurosurgery & Anesthesia & intensive care medicine, Karolinska University Hospital, Stockholm, Sweden
[15] NIHR Surgical Reconstruction and Microbiology Research Centre, Birmingham, UK
[16] Anesthesie-Réanimation, Assistance Publique – Hopitaux de Paris, Paris, France
[17] Department of Anesthesia & ICU, AOU Città della Salute e della Scienza di Torino - Orthopedic and Trauma Center, Torino, Italy
[18] Department of Neurology, Odense University Hospital, Odense, Denmark
[19] BehaviourWorks Australia, Monash Sustainability Institute, Monash University, Victoria, Australia
[20] Department of Public Health, Faculty of Health Sciences and Social Work, Trnava University, Trnava, Slovakia
[21] Quesgen Systems Inc., Burlingame, California, USA
[22] Australian & New Zealand Intensive Care Research Centre, Department of Epidemiology and Preventive Medicine, School of Public Health and Preventive Medicine, Monash University, Melbourne, Australia
[23] Department of Surgery and Perioperative Science, Umeå University, Umeå, Sweden
[24] Department of Neurosurgery, Medical School, University of Pécs, Hungary and Neurotrauma Research Group, János Szentágothai Research Centre, University of Pécs, Hungary
[25] Department of Medical Psychology, Universitätsklinikum Hamburg-Eppendorf, Hamburg, Germany
[26] Brain Physics Lab, Division of Neurosurgery, Dept of Clinical Neurosciences, University of Cambridge, Addenbrooke's Hospital, Cambridge, UK
[27] Neuro ICU, Fondazione IRCCS Cà Granda Ospedale Maggiore Policlinico, Milan, Italy
[28] ANZIC Research Centre, Monash University, Department of Epidemiology and Preventive Medicine, Melbourne, Victoria, Australia
[29] Department of Neurosurgery, Hospital of Cruces, Bilbao, Spain
[30] NeuroIntensive Care, Niguarda Hospital, Milan, Italy
[31] School of Medicine and Surgery, Università Milano Bicocca, Milano, Italy
[32] NeuroIntensive Care, ASST di Monza, Monza, Italy
[33]Department of Neurosurgery, Medical Faculty RWTH Aachen University, Aachen, Germany
[34] Department of Anesthesiology and Intensive Care Medicine, University Hospital Bonn, Bonn, Germany
[35] Department of Anesthesia & Neurointensive Care, Cambridge University Hospital NHS Foundation Trust, Cambridge, UK

[36] School of Public Health & PM, Monash University and The Alfred Hospital, Melbourne, Victoria, Australia
[37] Radiology/MRI department, MRC Cognition and Brain Sciences Unit, Cambridge, UK
[38] Institute of Medical Psychology and Medical Sociology, Universitätsmedizin Göttingen, Göttingen, Germany
[39] Oxford University Hospitals NHS Trust, Oxford, UK
[40] Intensive Care Unit, CHU Poitiers, Potiers, France
[41] University of Manchester NIHR Biomedical Research Centre, Critical Care Directorate, Salford Royal Hospital NHS Foundation Trust, Salford, UK
[42] Movement Science Group, Faculty of Health and Life Sciences, Oxford Brookes University, Oxford, UK
[43] Department of Neurosurgery, Antwerp University Hospital and University of Antwerp, Edegem, Belgium
[44] Department of Anesthesia & Intensive Care, Maggiore Della Carità Hospital, Novara, Italy
[45] Department of Neurosurgery, University Hospitals Leuven, Leuven, Belgium
[46] Department of Neurosurgery, Clinical centre of Vojvodina, Faculty of Medicine, University of Novi Sad, Novi Sad, Serbia
[47] Division of Anaesthesia, University of Cambridge, Addenbrooke's Hospital, Cambridge, UK
[48] Center for Stroke Research Berlin, Charité – Universitätsmedizin Berlin, corporate member of Freie Universität Berlin, Humboldt-Universität zu Berlin, and Berlin Institute of Health, Berlin, Germany
[49] Intensive Care Unit, CHR Citadelle, Liège, Belgium
[50] Department of Anaesthesiology and Intensive Therapy, University of Pécs, Pécs, Hungary
[51] Departments of Neurology, Clinical Neurophysiology and Neuroanesthesiology, Region Hovedstaden Rigshospitalet, Copenhagen, Denmark
[52] National Institute for Stroke and Applied Neurosciences, Faculty of Health and Environmental Studies, Auckland University of Technology, Auckland, New Zealand
[53] Department of Neurology, Erasmus MC, Rotterdam, the Netherlands
[54] Department of Anesthesiology and Intensive care, University Hospital Northern Norway, Tromso, Norway
[55] Department of Neurosurgery, Hadassah-hebrew University Medical center, Jerusalem, Israel
[56] Fundación Instituto Valenciano de Neurorrehabilitación (FIVAN), Valencia, Spain
[57] Department of Neurosurgery, Shanghai Renji hospital, Shanghai Jiaotong University/school of medicine, Shanghai, China
[58] Karolinska Institutet, INCF International Neuroinformatics Coordinating Facility, Stockholm, Sweden
[59] Emergency Department, CHU, Liège, Belgium
[60] Neurosurgery clinic, Pauls Stradins Clinical University Hospital, Riga, Latvia
[61] Department of Computing, Imperial College London, London, UK
[62] Department of Neurosurgery, Hospital Universitario 12 de Octubre, Madrid, Spain
[63] Department of Anesthesia, Critical Care and Pain Medicine, Medical University of Vienna, Austria
[64] Department of Public Health, Erasmus Medical Center-University Medical Center, Rotterdam, The Netherlands
[65] College of Health and Medicine, Australian National University, Canberra, Australia
[66] Department of Neurosurgery, Neurosciences Centre & JPN Apex trauma centre, All India Institute of Medical Sciences, New Delhi-110029, India
[67] Department of Neurosurgery, Erasmus MC, Rotterdam, the Netherlands
[68] Department of Neurosurgery, Oslo University Hospital, Oslo, Norway
[69] Division of Psychology, University of Stirling, Stirling, UK
[70] Division of Neurosurgery, Department of Clinical Neurosciences, Addenbrooke's Hospital & University of Cambridge, Cambridge, UK

[71] Department of Neurology, University of Groningen, University Medical Center Groningen, Groningen, Netherlands
[72] Neurointensive Care , Sheffield Teaching Hospitals NHS Foundation Trust, Sheffield, UK
[73] Salford Royal Hospital NHS Foundation Trust Acute Research Delivery Team, Salford, UK
[74] Department of Intensive Care and Department of Ethics and Philosophy of Medicine, Erasmus Medical Center, Rotterdam, The Netherlands
[75] Department of Clinical Neuroscience, Neurosurgery, Umeå University, Umeå, Sweden
[76] Hungarian Brain Research Program - Grant No. KTIA_13_NAP-A-II/8, University of Pécs, Pécs, Hungary
[77] Department of Anaesthesiology, University Hospital of Aachen, Aachen, Germany
[78] Cyclotron Research Center , University of Liège, Liège, Belgium
[79] Centre for Urgent and Emergency Care Research (CURE), Health Services Research Section, School of Health and Related Research (ScHARR), University of Sheffield, Sheffield, UK
[80] Emergency Department, Salford Royal Hospital, Salford UK
[81] Institute of Research in Operative Medicine (IFOM), Witten/Herdecke University, Cologne, Germany
[82] VP Global Project Management CNS, ICON, Paris, France
[83] Department of Anesthesiology-Intensive Care, Lille University Hospital, Lille, France
[84] Department of Neurosurgery, Rambam Medical Center, Haifa, Israel
[85] Department of Anesthesiology & Intensive Care, University Hospitals Southhampton NHS Trust, Southhampton, UK
[86] Cologne-Merheim Medical Center (CMMC), Department of Traumatology, Orthopedic Surgery and Sportmedicine, Witten/Herdecke University, Cologne, Germany
[87] Intensive Care Unit, Southmead Hospital, Bristol, Bristol, UK
[88] Department of Neurological Surgery, University of California, San Francisco, California, USA
[89] Department of Anesthesia & Intensive Care,M. Bufalini Hospital, Cesena, Italy
[90] Department of Neurosurgery, University Hospital Heidelberg, Heidelberg, Germany
[91] Department of Neurosurgery, The Walton centre NHS Foundation Trust, Liverpool, UK
[92] Department of Medical Genetics, University of Pécs, Pécs, Hungary
[93] Department of Neurosurgery, Emergency County Hospital Timisoara , Timisoara, Romania
[94] School of Medical Sciences, Örebro University, Örebro, Sweden
[95] Institute for Molecular Medicine Finland, University of Helsinki, Helsinki, Finland
[96] Analytic and Translational Genetics Unit, Department of Medicine; Psychiatric & Neurodevelopmental Genetics Unit, Department of Psychiatry; Department of Neurology, Massachusetts General Hospital, Boston, MA, USA
[97] Program in Medical and Population Genetics; The Stanley Center for Psychiatric Research, The Broad Institute of MIT and Harvard, Cambridge, MA, USA
[98] Department of Radiology, University of Antwerp, Edegem, Belgium
[99] Department of Anesthesiology & Intensive Care, University Hospital of Grenoble, Grenoble, France
[100] Department of Anesthesia & Intensive Care, Azienda Ospedaliera Università di Padova, Padova, Italy
[101] Dept. of Neurosurgery, Leiden University Medical Center, Leiden, The Netherlands and Dept. of Neurosurgery, Medical Center Haaglanden, The Hague, The Netherlands
[102] Department of Neurosurgery, Helsinki University Central Hospital
[103] Division of Clinical Neurosciences, Department of Neurosurgery and Turku Brain Injury Centre, Turku University Hospital and University of Turku, Turku, Finland
[104] Department of Anesthesiology and Critical Care, Pitié -Salpêtrière Teaching Hospital, Assistance Publique, Hôpitaux de Paris and University Pierre et Marie Curie, Paris, France
[105] Neurotraumatology and Neurosurgery Research Unit (UNINN), Vall d'Hebron Research Institute, Barcelona, Spain

[106] Department of Neurosurgery, Kaunas University of technology and Vilnius University, Vilnius, Lithuania
[107] Department of Neurosurgery, Rezekne Hospital, Latvia
[108] Department of Anaesthesia, Critical Care & Pain Medicine NHS Lothian & University of Edinburg, Edinburgh, UK
[109] Director, MRC Biostatistics Unit, Cambridge Institute of Public Health, Cambridge, UK
[110] Department of Physical Medicine and Rehabilitation, Oslo University Hospital/University of Oslo, Oslo, Norway
[111] Division of Orthopedics, Oslo University Hospital, Oslo, Norway
[112] Institue of Clinical Medicine, Faculty of Medicine, University of Oslo, Oslo, Norway
[113] Broad Institute, Cambridge MA Harvard Medical School, Boston MA, Massachusetts General Hospital, Boston MA, USA
[114] National Trauma Research Institute, The Alfred Hospital, Monash University, Melbourne, Victoria, Australia
[115] Department of Neurosurgery, Odense University Hospital, Odense, Denmark
[116] International Neurotrauma Research Organisation, Vienna, Austria
[117] Klinik für Neurochirurgie, Klinikum Ludwigsburg, Ludwigsburg, Germany
[118] Division of Biostatistics and Epidemiology, Department of Preventive Medicine, University of Debrecen, Debrecen, Hungary
[119] Department Health and Prevention, University Greifswald, Greifswald, Germany
[120] Department of Anaesthesiology and Intensive Care, AUVA Trauma Hospital, Salzburg, Austria
[121] Department of Neurology, Elisabeth-TweeSteden Ziekenhuis, Tilburg, the Netherlands
[122] Department of Neuroanesthesia and Neurointensive Care, Odense University Hospital, Odense, Denmark
[123] Department of Neuromedicine and Movement Science, Norwegian University of Science and Technology, NTNU, Trondheim, Norway
[124] Department of Physical Medicine and Rehabilitation, St.Olavs Hospital, Trondheim University Hospital, Trondheim, Norway
[125] Department of Neurosurgery, University of Pécs, Pécs, Hungary
[126] Division of Neuroscience Critical Care, John Hopkins University School of Medicine, Baltimore, USA
[127] Department of Neuropathology, Queen Elizabeth University Hospital and University of Glasgow, Glasgow, UK
[128] Dept. of Department of Biomedical Data Sciences, Leiden University Medical Center, Leiden, The Netherlands
[129] Department of Pathophysiology and Transplantation, Milan University, and Neuroscience ICU, Fondazione IRCCS Cà Granda Ospedale Maggiore Policlinico, Milano, Italy
[130] Department of Radiation Sciences, Biomedical Engineering, Umeå University, Umeå, Sweden
[131] Perioperative Services, Intensive Care Medicine and Pain Management, Turku University Hospital and University of Turku, Turku, Finland
[132] Department of Neurosurgery, Kaunas University of Health Sciences, Kaunas, Lithuania
[133] Intensive Care and Department of Pediatric Surgery, Erasmus Medical Center, Sophia Children's Hospital, Rotterdam, The Netherlands
[134] Department of Neurosurgery, Kings college London, London, UK
[135] Neurologie, Neurochirurgie und Psychiatrie, Charité – Universitätsmedizin Berlin, Berlin, Germany
[136] Department of Intensive Care Adults, Erasmus MC– University Medical Center Rotterdam, Rotterdam, the Netherlands
[137] icoMetrix NV, Leuven, Belgium
[138] Movement Science Group, Faculty of Health and Life Sciences, Oxford Brookes University, Oxford, UK

[139] Psychology Department, Antwerp University Hospital, Edegem, Belgium
[140] Director of Neurocritical Care, University of California, Los Angeles, USA
[141] Department of Neurosurgery, St.Olavs Hospital, Trondheim University Hospital, Trondheim, Norway
[142] Department of Emergency Medicine, University of Florida, Gainesville, Florida, USA
[143] Department of Neurosurgery, Charité – Universitätsmedizin Berlin, corporate member of Freie Universität Berlin, Humboldt-Universität zu Berlin, and Berlin Institute of Health, Berlin, Germany
[144] VTT Technical Research Centre, Tampere, Finland
[145] Section of Neurosurgery, Department of Surgery, Rady Faculty of Health Sciences, University of Manitoba, Winnipeg, MB, Canada

Appendix 2: STROBE Statement

	Item No	Recommendation	Page No
**Title and abstract**	1	(*a*) Indicate the study's design with a commonly used term in the title or the abstract<br>(b) Provide in the abstract an informative and balanced summary of what was done and what was found	1
**Introduction**			
Background/rationale	2	Explain the scientific background and rationale for the investigation being reported	2
Objectives	3	State specific objectives, including any prespecified hypotheses	3
**Methods**			
Study design	4	Present key elements of study design early in the paper	3
Setting	5	Describe the setting, locations, and relevant dates, including periods of recruitment, exposure, follow-up, and data collection	3
Participants	6	(*a*) Give the eligibility criteria, and the sources and methods of selection of participants. Describe methods of follow-up<br>(b) For matched studies, give matching criteria and number of exposed and unexposed	3
Variables	7	Clearly define all outcomes, exposures, predictors, potential confounders, and effect modifiers. Give diagnostic criteria, if applicable	3-4, Supplemental Notes1
Data sources/ measurement	8*	For each variable of interest, give sources of data and details of methods of assessment (measurement). Describe comparability of assessment methods if there is more than one group	3-4, Supplemental Notes1
Bias	9	Describe any efforts to address potential sources of bias	4, Supplemental Table S1-S3

Study size	10	Explain how the study size was arrived at	Figure 1
Quantitative variables	11	Explain how quantitative variables were handled in the analyses. If applicable, describe which groupings were chosen and why	3-4, Supplemental Notes 2
Statistical methods	12	(*a*) Describe all statistical methods, including those used to control for confounding<br>(b) Describe any methods used to examine subgroups and interactions<br>(c) Explain how missing data were addressed<br>(d) If applicable, explain how loss to follow-up was addressed<br>(e) Describe any sensitivity analyses	4, Supplemental Notes 2
**Results**			
Participants	13*	(a) Report numbers of individuals at each stage of study—eg numbers potentially eligible, examined for eligibility, confirmed eligible, included in the study, completing follow-up, and analysed<br>(b) Give reasons for non-participation at each stage<br>(c) Consider use of a flow diagram	4, Figure 1
Descriptive data	14*	(a) Give characteristics of study participants (eg demographic, clinical, social) and information on exposures and potential confounders<br>(b) Indicate number of participants with missing data for each variable of interest<br>(c) Summarise follow-up time (eg, average and total amount)	5, Table 1, Supplemental Table S3-S7
Outcome data	15*	Report numbers of outcome events or summary measures over time	6-7, Table 2
Main results	16	(*a*) Give unadjusted estimates and, if applicable, confounder-adjusted estimates and their precision (eg, 95% confidence interval). Make clear which confounders were adjusted for and why they were included<br>(b) Report category boundaries when continuous variables were categorized<br>(c) If relevant, consider translating estimates of relative risk into absolute risk for a meaningful time period	7, Figure 2, Table 3-4, , Supplemental Notes 3, Supplemental Table S8
Other analyses	17	Report other analyses done—eg analyses of subgroups and interactions, and sensitivity analyses	8, Table 5

Discussion			
Key results	18	Summarise key results with reference to study objectives	8-10
Limitations	19	Discuss limitations of the study, taking into account sources of potential bias or imprecision. Discuss both direction and magnitude of any potential bias	10-11
Interpretation	20	Give a cautious overall interpretation of results considering objectives, limitations, multiplicity of analyses, results from similar studies, and other relevant evidence	8-10
Generalisability	21	Discuss the generalisability (external validity) of the study results	8-10
**Other information**			
Funding	22	Give the source of funding and the role of the funders for the present study and, if applicable, for the original study on which the present article is based	Acknowledgement

Supplemental References

1. Teasdale G, Maas A, Lecky F, Manley G, Stocchetti N, Murray G. The Glasgow Coma Scale at 40 years: standing the test of time. *The Lancet Neurology*. 2014;13(8):844-854.
2. Marmarou A, Lu J, Butcher I, et al. Prognostic value of the Glasgow Coma Scale and pupil reactivity in traumatic brain injury assessed pre-hospital and on enrollment: an IMPACT analysis. *Journal of neurotrauma*. 2007;24(2):270-280.
3. Wodzin TAGE. *The Abbreviated Injury Scale 2005. Update 2008*. American Association for Automotive Medicine (AAAM); 2008.
4. van Leeuwen N, Lingsma HF, Perel P, et al. Prognostic value of major extracranial injury in traumatic brain injury: an individual patient data meta-analysis in 39,274 patients. *Neurosurgery*. Apr 2012;70(4):811-8; discussion 818. doi:10.1227/NEU.0b013e318235d640
5. Copes WS, Champion HR, Sacco WJ, Lawnick MM, Keast SL, Bain LW. The injury severity score revisited. *Journal of Trauma and Acute Care Surgery*. 1988;28(1):69-77.
6. Sewalt C, Venema E, Wiegers E, et al. Trauma models to identify major trauma and mortality in the prehospital setting. *The British journal of surgery*. 2020;107(4):373.
7. Wilson JL, Pettigrew LE, Teasdale GM. Structured interviews for the Glasgow Outcome Scale and the extended Glasgow Outcome Scale: guidelines for their use. *Journal of neurotrauma*. 1998;15(8):573-585.
8. Wilson J, Edwards P, Fiddes H, Stewart E, Teasdale GM. Reliability of postal questionnaires for the Glasgow Outcome Scale. *Journal of neurotrauma*. 2002;19(9):999-1005.
9. Bollen KA. Structural equation models. *Encyclopedia of biostatistics*. 2005;7
10. Little TD, Preacher KJ, Selig JP, Card NA. New developments in latent variable panel analyses of longitudinal data. *International journal of behavioral development*. 2007;31(4):357-365.
11. Liu Y, Millsap RE, West SG, Tein J-Y, Tanaka R, Grimm KJ. Testing measurement invariance in longitudinal data with ordered-categorical measures. *Psychological Methods*. 2017;22(3):486.

12. Bentler PM. Comparative fit indexes in structural models. *Psychological bulletin*. 1990;107(2):238.
13. Browne MW, Cudeck R. Alternative ways of assessing model fit. *Sociological methods & research*. 1992;21(2):230-258.
14. Chen FF. Sensitivity of goodness of fit indexes to lack of measurement invariance. *Structural Equation Modeling: A Multidisciplinary Journal*. 2007;14(3):464-504.
15. Curran PJ, Bollen KA. The best of both worlds: Combining autoregressive and latent curve models. 2001;
16. Masten AS, Cicchetti D. Developmental cascades. *Development and psychopathology*. 2010;22(3):491-495.
17. Selig JP, Little TD. Autoregressive and cross-lagged panel analysis for longitudinal data. 2012;
18. Kearney MW. Cross lagged panel analysis. *The SAGE encyclopedia of communication research methods*. 2017:312-314.
19. Brown TA. *Confirmatory factor analysis for applied research*. Guilford publications; 2015.
20. Kline RB. *Principles and practice of structural equation modeling*. Guilford publications; 2015.
21. Tucker LR, Lewis C. A reliability coefficient for maximum likelihood factor analysis. *Psychometrika*. 1973;38(1):1-10.
22. Hu Lt, Bentler PM. Cutoff criteria for fit indexes in covariance structure analysis: Conventional criteria versus new alternatives. *Structural equation modeling: a multidisciplinary journal*. 1999;6(1):1-55.
